# Genome-wide identification of CCT genes in wheat (*Triticum aestivum* L.) and their expression analysis during vernalization

**DOI:** 10.1371/journal.pone.0262147

**Published:** 2022-01-05

**Authors:** HongWei Zhang, Bo Jiao, FuShuang Dong, XinXia Liang, Shuo Zhou, HaiBo Wang

**Affiliations:** 1 College of Agronomy, Hebei Agricultural University, Baoding, Hebei, China; 2 Plant Genetic Engineering Center of Hebei Province, Institute of Biotechnology and Food Science, Hebei Academy of Agriculture and Forestry Sciences, Shijiazhuang, Hebei, China; Institute of Genetics and Developmental Biology Chinese Academy of Sciences, CHINA

## Abstract

Numerous CCT genes are known to regulate various biological processes, such as circadian rhythm regulation, flowering, light signaling, plant development, and stress resistance. The CCT gene family has been characterized in many plants but remains unknown in the major cereal wheat (*Triticum aestivum L*.). Extended exposure to low temperature (vernalization) is necessary for winter wheat to flower successfully. *VERNALIZATION2* (*VRN2*), a specific CCT-containing gene, has been proved to be strongly associated with vernalization in winter wheat. Mutation of all *VRN2* copies in three subgenomes results in the eliminated demands of low temperature in flowering. However, no other CCT genes have been reported to be associated with vernalization to date. The present study screened CCT genes in the whole wheat genome, and preliminarily identified the vernalization related CCT genes through expression analysis. 127 CCT genes were identified in three subgenomes of common wheat through a hidden Markov model-based method. Based on multiple alignment, these genes were grouped into 40 gene clusters, including the duplicated gene clusters *TaCMF6* and *TaCMF8*, each tandemly arranged near the telomere. The phylogenetic analysis classified these genes into eight groups. The transcriptome analysis using leaf tissues collected before, during, and after vernalization revealed 49 upregulated and 31 downregulated CCT genes during vernalization, further validated by quantitative real-time PCR. Among the differentially expressed and well-investigated CCT gene clusters analyzed in this study, *TaCMF11*, *TaCO18*, *TaPRR95*, *TaCMF6*, and *TaCO16* were induced during vernalization but decreased immediately after vernalization, while *TaCO1*, *TaCO15*, *TaCO2*, *TaCMF8*, and *TaPPD1* were stably suppressed during and after vernalization. These data imply that some vernalization related CCT genes other than *VRN2* may exist in wheat. This study improves our understanding of CCT genes and provides a foundation for further research on CCT genes related to vernalization in wheat.

## Introduction

Timing of flowering is a crucial agronomic trait that determines the environmental adaptability and grain yield in plants [1]. Plants integrate exogenous signals, such as photoperiod and winter temperature (vernalization), and endogenous signals such as autonomous pathway to modulate flowering time [2]. The successful transition from vegetative stage to reproductive stage thus occurs in the most proper time. Several genes which harbor a unique CCT domain, are involved in these regulatory pathways. The CCT domain, initially described in the *Arabidopsis* protein CONSTANS (CO), CONSTANS-LIKE (COL), and TIMING OF CAB EXPRESSION 1 (TOC1), is about 43 amino acids long and has a putative nuclear localization signal at the N-terminal [3]. The proteins with the CCT domain have been named as CCT proteins and divided into four subfamilies based on the additional domains. These four subfamilies are CCT MOTIF FAMILY (CMF), CO/COL, PSEUDO-RESPONSE REGULATOR (PRR), and ZINC-FINGER PROTEIN EXPRESSED IN INFLORESCENCE MERISTEM (ZIM). CMF proteins do not harbor any characterized domains other than the CCT domain; CO/COL proteins harbor one or two additional B-box domains; PRR proteins have Response_reg domain [4]; ZIM proteins, also referred to as CMF proteins in some works, harbor three domains: tify, CCT, and ZnF_GATA [5–8].

The CCT genes have been proved to be associated with several biological processes, such as circadian rhythm regulation and flowering [2]. TOC1, a central component of the circadian rhythm, interacts with LATE ELONGATED HYPOCOTYLL (LHY) and CIRCADIAN CLOCK-ASSOCIATED 1 (CCA1) to form a complex feedback loop in *Arabidopsis* [9,10]. A mutation in this gene (*toc1-1*) resulted in a shorter circadian rhythm and different photoperiodic flowering responses relative to the wild-type plants [3]. PRR9/PRR7/PRR5 and CCA1/LHY suppress the expression of *CYCLING DOF FACTOR 1* (*CDF1*) and *GIGANTEA* (*GI*), respectively. Circadian rhythm signals are then transmitted to *CO* via its inhibition by CDF1 and promotion by GI [11]. The oscillatory expression of *CO* integrates different photoperiod, and results in the divergence of flowering patterns in different plants. The highest mRNA abundance of *CO* under long-day (LD) conditions is most pronounced in the late afternoon [12,13], while the expression of its ortholog peaks during the dark period under short-day (SD) conditions [14]. Moreover, CO protein is stabilized under light but degraded in the dark. These result in the accumulation of CO proteins in LD plants and induces flowering, but deficiency of CO proteins in SD plants [13,15]. Alternatively, a B-type response regulator EARLY HEADING DATE 1 (Ehd1) induced *FLOWER LOCUS T-LIKE* (*FT-like*) genes to promote flowering under SD conditions in rice, a SD type model species [16].

In *Arabidopsis*, CO can replace HEME ACTIVATOR PROTEIN 2 (HAP2) to form a trimeric CO/HAP3/HAP5 complex that binds to *FT* promoter and induces its expression [17,18]. The subsequent induction of *SUPPRESSOR OF OVER-EXPRESSION OF CONSTANS 1* (*SOC1*) by FT promotes flowering under LD conditions [19]. Similarly, a trimeric complex composed of CO1/CO2, HAP3, and HAP5 directly binds to *VERNALIZATION 3* (*VRN3*, the ortholog of *FT*) promoter and induces its expression under LD conditions, and flowering is induced in wheat [20,21]. However, VRN2, a flowering repressor, competes with CO2 and represses the expression of *VRN3*, leading to the inhibition of flowering [21]. Thus, only when *VRN2* expression is wholly inhibited, the *VRN3* gene gets successfully induced, and flowering is subsequently promoted in wheat. The wheat *VRN2* is induced under LD conditions, similar to *GRAIN NUNBER*, *PLANT HEIGHT AND HEADING DATE 7* (*Ghd7*, the ortholog of *VRN2*) in rice, and therefore, flowering does not occur before vernalization [22–24]. *VRN2* is suppressed during vernalization directly, and indirectly by the upregulation of *VRN1* [25,26]. The suppression of *VRN2* then eliminates the inhibition of flowering in winter wheat after vernalization. Interestingly, instead of *CO* in *Arabidopsis*, *PHOTOPERIOD 1* (*PPD1*) is thought to be the most important photoperiod related genes to promote flower in wheat [27]. Its loss-of-function mutant inhibits flowering and gain-of-function mutant promotes flowering. In these processes, the elevated *PPD1* expression correlates with the upregulation of *VRN3*, and vice versa [28–30].

In summary, the CCT genes are widely associated with circadian rhythm and photoperiod flowering. At least 19 of the 41 characterized rice CCT genes have been associated with heading date [7,31,32]. Studies have also reported *VRN2* as a critical flowering suppressor gene associated with vernalization in cereals [22], suggesting the role of some other CCT genes in flowering during vernalization in wheat. So far, CCT proteins have been studied via whole-genome analysis in monocot and dicot plants, such as *sorghum*, *Setaria italica*, *Brachypodium* [4], *Oryza sativa*, *Hordeum vulgare*, *Arabidopsis* [4,33], *Aegilops* [34], and *Medicago* [1] but not in wheat. The present study performed a whole-genome analysis of CCT domain-containing genes in wheat employing a hidden Markov model (HMM)-based method using HMMER v3.0. Further, the sequence and structure information, phylogenetic relationship, and chromosomal location of the identified genes and their expression patterns before, during, and after vernalization were investigated. Our findings will improve the understanding of wheat CCT genes and their relationship with vernalization and provide the foundation for further research on wheat vernalization.

## Materials and methods

### Identification of wheat CCT genes

The potential CCT genes in wheat were identified following the method of Zhan et al. [35], with minor modifications. The HMM for the CCT domain (PF06203) was obtained from Pfam v34.0 (http://pfam.xfam.org/browse), and the available protein sequences and structure information of the wheat cultivar Chinese Spring (CS), *Brachypodium*, and *Arabidopsis* (Release 47) were downloaded from the Ensembl Plants portal (http://plants.ensembl.org/info/data/ftp/index.html). The available rice proteins (Release 7) were downloaded from the Rice Genome Annotation Project (RGAP, http://rice.plantbiology.msu.edu/). Subsequently, the CCT proteins of these four species were obtained using the hmmsearch command embedded in HMMER v3.0 according to the HMM and available sequences. After filtering out the redundant sequences, the CCT proteins were confirmed using the Batch CD-Search tool in National Center for Biotechnology Information (NCBI, https://www.ncbi.nlm.nih.gov/cdd) with default criteria and were designated by comparing with the available sequences in *Brachypodium*, rice, and wheat. The controversial sequences on splicing sites were reassessed using FGENESH+ (http://linux1.softberry.com/), and the genes from different subgenomes were complemented using BLAST.

### Phylogenetic analysis

The identified CCT proteins of wheat, rice, *Brachypodium*, and *Arabidopsis* were aligned using Clustal Omega (https://www.ebi.ac.uk/Tools/msa/clustalo/). The phylogenetic analysis was performed with these aligned proteins using MEGA v6.0 following the Unweighted Pair Group Method with Arithmetic mean (UPGMA) method [36], using 1000 bootstrap replications and Jones-Talor-Thornton (JTT) model. The generated phylogenetic tree was reconfigured and visualized by iTOL v4 (https://itol.embl.de/) [37]. The phylogenetic tree of wheat CCT proteins displayed in gene structure visualization and heat map was constructed using the same method.

### Protein properties and sequence analysis

The theoretical isoelectric point and molecular weight of the identified CCT proteins were calculated using the Compute pI/Mw tool in ExPASy (https://web.expasy.org/compute_pi/). Subcellular localization of these proteins was performed using CELLO v2.5 (http://cello.life.nctu.edu.tw/) [38]. The MEME suite (http://meme-suite.org/tools/meme) [39] and the Batch CD-Search tool [40] predicted the conserved motifs and domains of the proteins, respectively. The multiple alignment of CCT domains was shaded by BoxShade (https://embnet.vital-it.ch/software/BOX_form.html). Amino acids of the domains were represented using WebLogo (http://weblogo.berkeley.edu/logo.cgi). Visualization of gene structure and motifs and chromosomal location were performed by TBtools [41].

### Sample preparation and RNA extraction

The seeds of the winter wheat cultivar Shiluan02-1 kept in our laboratory were sown in a matrix (a mixture of nutrient soil and vermiculite with volume ratio of 1:1) and maintained for growth at 22°C under LD conditions (16 h/8 h) in a greenhouse. After two weeks, the leaves were collected just before the third leaf emerged (before vernalization, v0). The remaining plants were vernalized for six weeks at 4°C, followed by growth at 22°C under LD conditions. Two newly expanded leaves were collected every week during and after vernalization (v1, v2, v3, v4, v5 and v6 represented vernalization for one, two, three, four, five and six weeks, respectively; pv1 represented one week’s normal growth after vernalization). The samples were immediately frozen in liquid nitrogen and stored at -80°C. Triplicate samples were collected for each sampling stage, with 20 individual plants per sample.

RNA was extracted from the leaf samples using RNAprep Pure Plant Kit (Tiangen, Beijing, China) following the manufacturer’s instructions. Subsequently, RNA integrity was assessed by agarose gel electrophoresis, and RNA concentration was determined using NanoDrop2000 (Thermo Scientific, USA).

### Transcriptome sequencing

The library was constructed using NEBNext® Ultra^TM^ RNA Library Prep Kit (NEB, USA) following the manufacturer’s instructions, with minor modifications. Briefly, the mRNA was isolated from total RNA using VAHTS mRNA Capture Beads (Vazyme Biotech, Nanjing, China). The purification of the double-stranded cDNA and PCR products, as well as the fragment selection of ligation products were performed using VAHTS DNA Clean Beads (Vazyme Biotech, Nanjing, China). The constructed library was sequenced on a HiSeq 4000 System (Illumina, USA).

The quality of the sequenced data was evaluated using FastQC v0.11.8. The adaptor and low-quality reads were filtered from the raw reads using Trimmomatic v0.39. The generated clean reads were then mapped to the CS reference genome using HISAT v2.2.0 with default parameters. The expression abundance of the transcripts was calculated using StringTie v2.1.3, and measured as Fragments Per Kilobase of transcript per Million read pairs (FPKM). DESeq2 analyzed the Differentially Expressed Genes (DEGs). Visualization of expression profiling (heat map) and UpSet plot diagram were performed by TBtools [41]. The expression data of CCT genes were listed in S5 Table, and the differentially expressed CCT genes were listed in S3 Table.

### Quantitative real-time PCR analysis

One microgram of total RNA extracted from each leaf sample was used as the template to synthesize the first-strand cDNA using PrimeScript^TM^ RT Reagent Kit with gDNA Eraser (Perfect Real-Time) (TaKaRa, Japan). To further validate the expression profile of CCT genes obtained via transcriptome analysis, quantitative real-time PCR of the seven selected DEG clusters (*TaCMF6*, *TaCMF11*, *TaCO16*, *TaCO18*, *TaPRR95*, *TaCO1*, and *TaCO15*) and five most popularly studied CCT clusters (*TaZIM4*, *TaPPD1*, *TaCO2*, *TaTOC1*, and *TaCMF8*) was performed on ABI 7500 Real-Time PCR System (Thermo Scientific, USA). Gene expression was normalized to the reference gene *TaActin*, and the relative gene expression level was analyzed by the 2^-ΔΔCT^ method [42]. The primers used for the quantitative real-time PCR were listed in S4 Table.

## Results

### Wheat genome contains 127 CCT genes belonging to four subfamilies

Based on the available genome-wide assembly and annotation of wheat cultivar CS [43], wheat CCT genes were screened against their unique HMM (for the CCT domain) using the HMMER3.0 package. After redundant sequence filtering, Batch CD-Search, FGENESH+ verification, and gene complementation by BLAST, a total of 127 candidate sequences with CCT domain were obtained. These genes were subsequently designated based on the available CCT genes of *Brachypodium*, rice, and wheat [4,6]. For gene nomenclature, *TaCMF8-A2* indicated the second copy of the eighth *CMF* gene in the *T*. *aestivum* A genome. As a result, a total of 54 CO/COL genes, 46 CMF genes, 15 PRR genes, and 12 ZIM genes belonging to 18 CO/COL clusters, 13 CMF clusters, 5 PRR clusters, and 4 ZIM clusters were identified in wheat. Most of the designated CCT clusters contained three highly homologous members, each in a distinct subgenome. However, *TaCMF6* contained seven members, while *TaCMF8* contained six members, with two or three members tandemly arranged in the same subgenome. The results also showed that wheat CCT proteins had their amino acid residue lengths from 170 (TaCMF14-D) to 763 (TaPRR73-D). The molecular weight of these proteins was simultaneously predicted to be ranged from 19.48 kD (TaCMF14-D) to 83.21 kD (TaPRR73-D), and the theoretical isoelectric point from 4.32 (TaCO13-B) to 10.22 (TaCMF14-B). The majority of these proteins were predicted to be located in the nucleus (111 proteins), while 11 were found in the chloroplast, three in the cytoplasm, and two in the extracellular matrix. These observations imply that CCT proteins are predominantly nuclear proteins (S1 Table) [21].

### CCT proteins belong to eight well-defined groups

**Fig 1:**
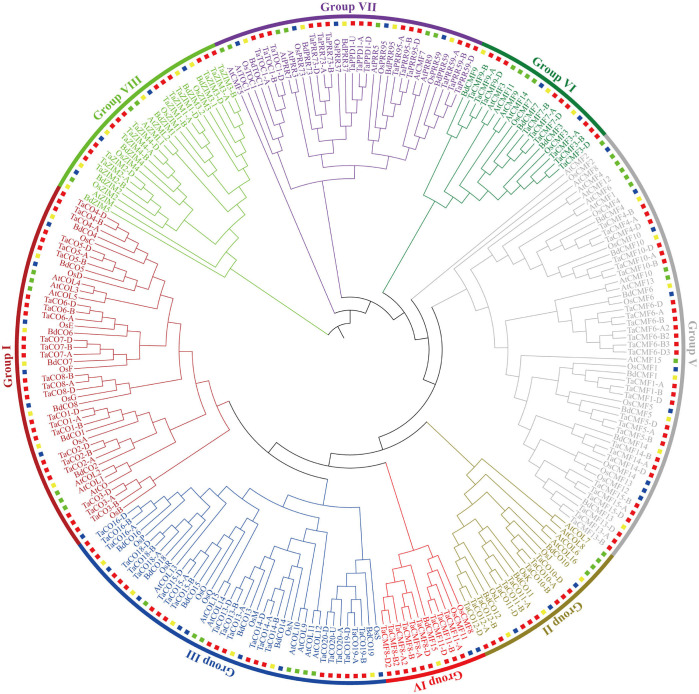
Phylogenetic analysis of CCT proteins in wheat, *Arabidopsis*, *Brachypodium*, and rice. The CCT proteins were phylogenetically analyzed using MEGA v6.0 following the UPGMA method. The proteins from different plants are represented in different colored squares (wheat, red; *Arabidopsis*, green; *Brachypodium*, yellow; rice, blue). The gene IDs are listed in S1 and S2 Tables.

Phylogenetic analysis of CCT proteins from wheat, *Arabidopsis*, *Brachypodium*, and rice revealed eight well-defined groups. Similar to the classification of CCT genes in previous studies, Groups I to III contained all the CO/COL proteins [33], while Groups IV to VI had most of the CMF proteins. TaCMF8 (also known as TaVRN2, a member of Group IV proteins), initially grouped within the COL family [4,33], was classified as a CMF protein in this study and a previous study due to the lack of B-box domain [4]. Interestingly, no *Arabidopsis* member was assigned to Group IV. Besides, together with AtCMF5 and AtCMF7, the PRR proteins were classified to Group VII, which was further divided into three clades. Group VIII contained all the ZIM proteins, and was further divided into two subgroups (Fig 1).

### Gene and protein structure of wheat CCTs differ considerably among the subfamilies

**Fig 2:**
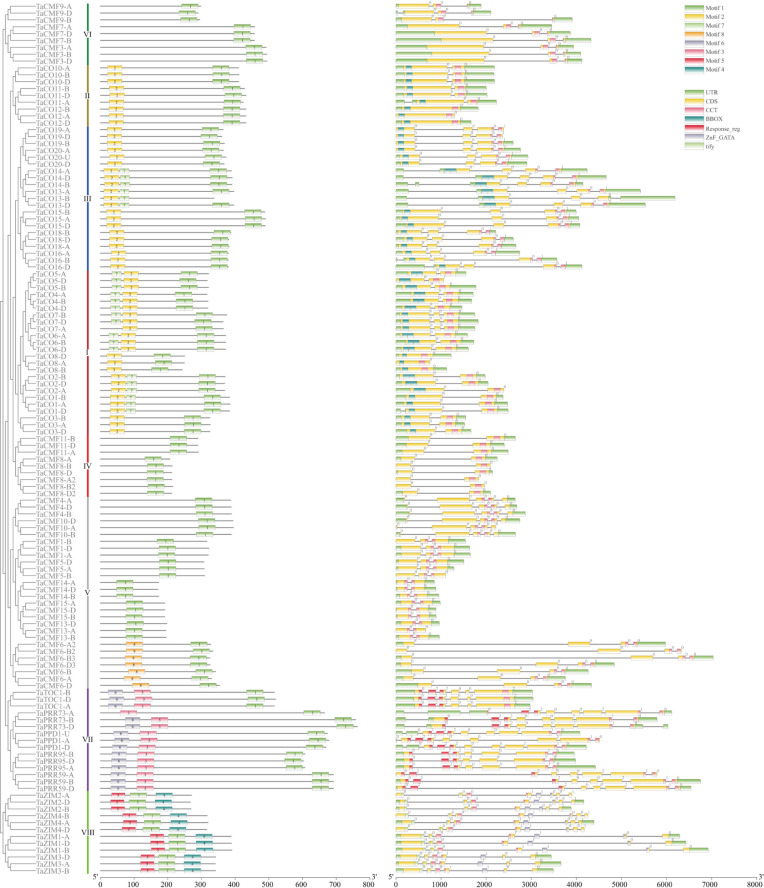
Phylogenetic analysis, conserved motif and structure analysis of CCT genes in wheat. The motifs and conserved domains were predicted using the MEME suite and the Batch CD-Search tool, respectively, and displayed using TBtools. Phylogenetic analysis of the proteins was performed using MEGA v6.0 following the UPGMA method.

In order to clarify the structural differences of CCT proteins in wheat, their motifs were analyzed using the MEME suite and conserved domains using the Batch CD-Search tool. The observations indicated that the classification of CCT proteins based on motifs and conserved domains were consistent with that based on phylogeny (Fig 2). Most of the CCT domains (corresponding to motif 1) were predicted to be located at the C-terminal of the protein, but TaCO13-B lacked this domain due to a premature transcriptional termination within the penultimate exon. ZIM proteins (Group VIII) were predicted to possess their CCT domains in the middle, with the extra tify domain (corresponding to motif 5) at the N-terminal and the Znf_GATA domain (corresponding to motif 4) at the C-terminal. Meanwhile, the PRR proteins (Group VII) were found to harbor their Response_reg domain (corresponding to motif 6 plus motif 3) at the N-terminal, and CO/COL proteins (Groups I to III) harbored one or two B-box zinc finger domains (corresponding to motif 2 or 7). Specifically, TaCO13 and TaCO14 possessed two B-boxes, while the remaining proteins of Group III and all proteins of Group II had only one; Group I contained both proteins with one and two B-boxes. Interestingly, all the CO/COL proteins having only one B-box domain contained B-box1 (motif 2), but not B-Box2 (motif 7). Meanwhile, the CMF proteins (Groups IV to VI) had no extra domains other than CCT, except that TaCMF6 in Group V possessed an unknown motif (motif 8) at its N-terminal.

**Fig 3:**
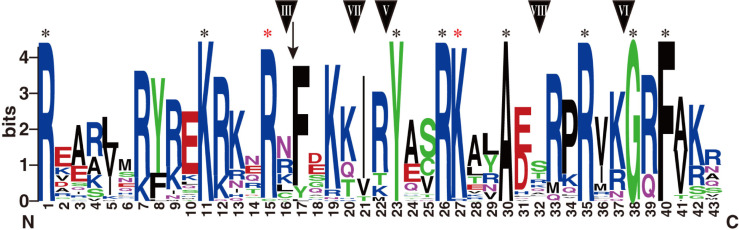
WebLogo representation of the conserved CCT domain. Black asterisks indicate identical residues in all CCT domains, and red asterisks indicate identical residues in most CCT domains. The black arrow indicates the residue insertion of PL before L17 in TaCMF7. Triangles with numbers representing the corresponding phylogenetic classification groups suggest the splitting site of the motif.

Furthermore, the conserved domains of these genes were sometimes split and located in tandem-arranged exons. More specifically, the tify domains were present in exons 1 and 2, and Zn_GATA domains in exons 5 and 6. Response_reg domains were located in the first three coding exons, whereas the B-box domains were always located in the first coding exon without splitting. In addition, the splitting of CCT domains was more complex, and their evolution always coincided with the phylogenetic of the genes. The ZIM genes (Group VIII) had the CCT domains in exons 3 and 4, and these domains were split at the 32^nd^ residue of the unique 43-amino acid peptides. The CCT domains of Group III proteins were split at the 16^th^ residue and that of Group V, VI, and VII proteins just after the 22^nd^, 37^th^, and 20^th^ residues, respectively. These genes harbored their CCT domains in the last two exons, except for *TaCMF4* and *TaCMF10* of Group V. These two clusters had their CCT domains in the antepenultimate and penultimate exons. Meanwhile, the CCT domain of *TaTOC1* of Group VII was present in the last exon without any splitting, consistent with the members of Groups I, II, and IV (Figs 2 and 3). Furthermore, the alignment of the CCT domains revealed a high conservation of these 43 amino acids in wheat: eight residues (R1, K11, Y23, R26, A30, R35, G38, and F40) were identical across all CCT proteins, whereas R15 and K27 were identical in CCT proteins except for TaCMF7 and TaCMF9, respectively. In addition, a PL insertion was present upstream of L17 in TaCMF7 (Fig 3, S1 Fig).

The analysis revealed a divergent exon distribution in these 127 genes, with 6, 42, 19, 27, 6, 3, 9, 12, and 3 genes harboring 1, 2, 3, 4, 5, 6, 7, 8, and 9 coding exons, respectively. Although most of Group I, II, and IV genes harbored two exons, *TaCO8* of Group I and *TaCO12* of Group II harbored only one. Genes of Group VI, together with *TaCO7* of Group I, had three exons; while genes of Group III, except for *TaCO13-B*, had four exons. Groups V (2 to 5 exons), VII (6 to 9 exons), and VIII (7 or 8 exons) genes had the most divergent exon distribution (Fig 2, S1 Table).

### Chromosomal location of wheat CCT genes reveals gene duplication and homologous recombination

**Fig 4:**
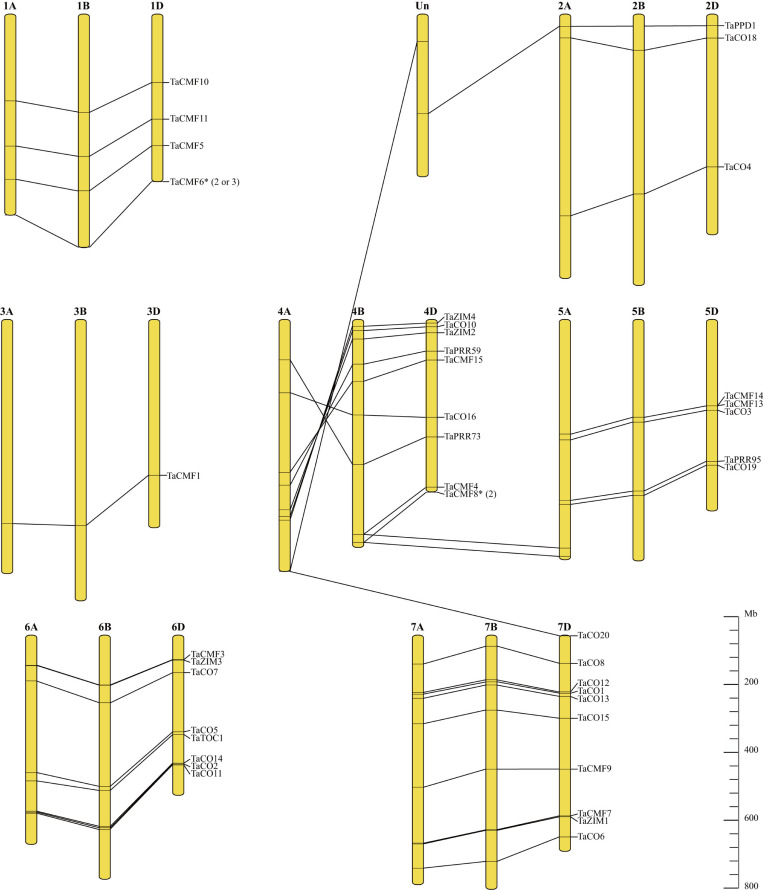
Chromosomal localization of CCT genes in wheat visualized by TBtools. Gene clusters with at least three genes distributed among the three subgenomes are marked on the right. Asterisks indicate the clusters with tandemly arranged genes, and the copy number in each subgenome is shown in parentheses.

The 127 CCT genes identified in this study were further analyzed for their distribution across the 21 chromosomes in wheat. Chromosome 3 contained the fewest CCT genes with only one in each subgenome, followed by chromosome 2 with three. Chromosomes 4B, 4D, and 7D had the highest number of CCT genes with 10 in each subgenome, followed by chromosomes 7A and 7B with 9. Most of the gene clusters contained three genes, each collinearly located in their corresponding subgenome. However, two or three copies of *TaCMF6* and *TaCMF8* were found tandemly arranged near telomeres in each subgenome. Although tandemly arranged in chromosome 5, *TaCMF13* and *TaCMF14* were not clustered together, as *TaCMF13* was more closely related to *TaCMF15* in chromosome 4 (Fig 1, 63.58% identity by pairwise alignment between *TaCMF13* and *TaCMF15*, and 43.37% between *TaCMF13* and *TaCMF14*). Furthermore, the gene arrangement on chromosome 4A was reversed from that on chromosomes 4B and 4D, and the transposition of *TaCMF4-A* and *TaCMF8-A* from chromosome 4A to chromosome 5A revealed a recent recombination between chromosomes 4A and 5A near telomeres. The existence of *TaPPD1-A*, *-D*, and *-U* implied that *TaPPD1-U* was the B-genome copy of *TaPPD1*, and the arrangement of *TaCO20* in the genome indicated the possibility of the B-genome localization of *TaCO20-U* and the occurrence of another recombination event (Fig 4).

### Expression pattern of wheat CCT genes varies greatly during vernalization

**Fig 5:**
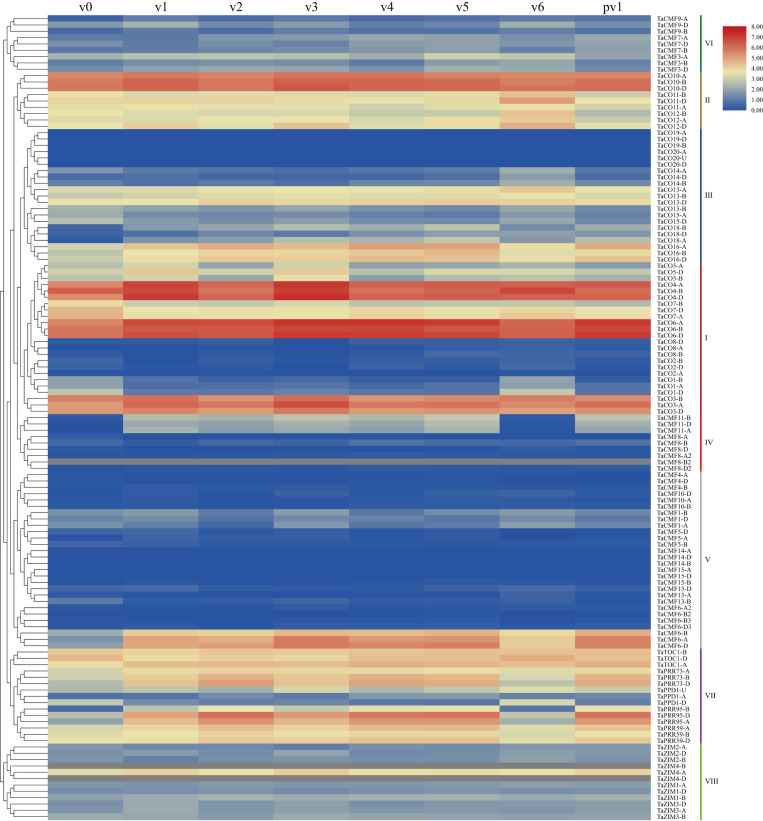
Expression profiling of CCT genes in wheat before, during, and after vernalization by RNA sequencing. The leaf samples were harvested before vernalization (v0), after one week’s vernalization (v1), two weeks’ vernalization (v2), three weeks’ vernalization (v3), four weeks’ vernalization (v4), five weeks’ vernalization (v5), six weeks’ vernalization (v6), and one-week normal growth after six weeks’ vernalization (pv1). The phylogenetic analysis that appeared here was the same as that in Fig 2.

A transcriptome analysis using leaf tissues of the winter wheat cultivar Shiluan 02–1 before, during, and after vernalization was performed to clarify the potential functions of CCT genes during vernalization in wheat. A total of 113, 110, 113, 106, 113, 113, 117, and 115 CCT genes were detected in pre-vernalized, vernalized for one, two, three, four, five, and six weeks, and post-vernalized samples, respectively. Among the 40 characterized CCT clusters (127 genes), *TaCO3*, *TaCO4*, *TaCO6*, and *TaCO10* were always expressed highly in these samples, but *TaCMF14*, *TaCO20*, *TaCMF15-A/D*, and *TaCO19-A/D* were undetected. Group II genes (CO/COL subfamily) exhibited a continuous high expression, but Group VI genes (CMF subfamily) exhibited a persistent low expression at all stages. *TaCO13*, *TaCO16*, and *TaCO15* of Group III (CO/COL subfamily) showed the highest expression before vernalization, in which *TaCO16* was upregulated while *TaCO15* was downregulated during vernalization. *TaCO18* of Group III was barely expressed before vernalization, but upregulated during vernalization. Group I genes (CO/COL subfamily) showed the greatest divergence in their expression: *TaCO2* and *TaCO8* showed almost no expression; *TaCO3*, *TaCO4*, and *TaCO6* showed the highest expression; but *TaCO1* showed moderate expression before vernalization. Among these clusters, *TaCO6* was upregulated but *TaCO1* was significantly downregulated during vernalization. Although six copies of *TaCMF8* were found in Group IV (CMF subfamily), only *TaCMF8-B* showed slight expression at all stages, as the expression data of *TaCMF8-B2* was deficient. Another cluster of Group IV, *TaCMF11*, was continuously upregulated during vernalization. Most clusters of Group V (CMF subfamily) exhibited continuous low expression except for the vernalization-induced cluster of *TaCMF6*, while the clusters of Group VII (PRR subfamily) always sustained its relatively high expression except for *TaPPD1*. The expression of *TaPRR95* and *TaPRR73* of Group VII were further increased during vernalization. Meanwhile, *TaZIM4-A* was the only Group VIII gene (ZIM subfamily) with a sustained high expression because the expression data of *TaZIM4-B/D* was also deficient (Fig 5).

**Fig 6:**
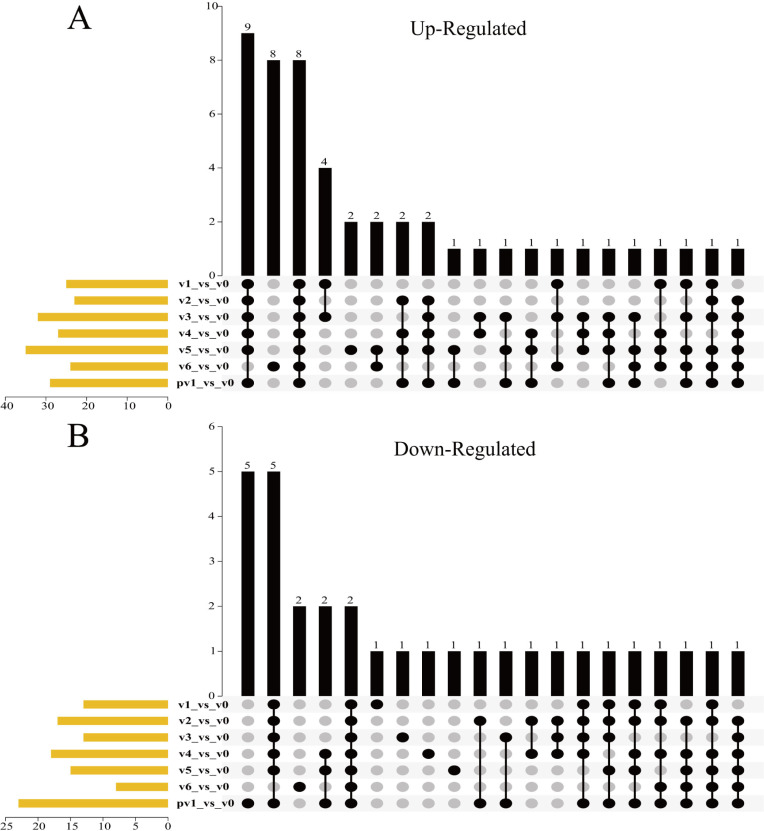
UpSet plot diagram of upregulated and downregulated CCT genes during/after vernalization compared with pre-vernalization. A: Upregulated genes; B: Downregulated genes. The leaf samples were harvested the same as those in Fig 5. Transcriptome sequencing was conducted to obtain the gene expression data, which was expressed as FPKM.

The expression of CCT genes during/after vernalization was then compared with those before vernalization. Among these, 49 genes were upregulated, and 31 were downregulated at least at a single time point. More specifically, compared to pre-vernalization, 25, 23, 32, 27, 35, 24, and 29 genes were upregulated, and 13, 17, 13, 18, 15, 8, and 23 genes were downregulated, after one, two, three, four, five, six weeks of vernalization, and post-vernalization (one-week normal growth after six weeks’ vernalization), respectively. Furthermore, compared to pre-vernalization, eight genes remained upregulated at all time points, eleven genes at six time points, but ten genes were upregulated at a single time point; on the contrary, two genes remained downregulated at all time points, seven genes at six time points, but eleven were downregulated at only a single time point. We measured the DEG ratios of 40 wheat CCT clusters. It was preliminarily considered that the clusters with a higher DEG ratio had relatively significant expression differences in comparison of during/after and pre-vernalization, and were more relevant to vernalization. We also defined the clusters with a DEG ratio greater than 60% as significant differentially expressed ones. As a result, *TaCMF11* (95.24% in DEG ratio), *TaCO18* (95.24%), *TaPRR95* (85.71%), *TaCO16* (80.95%), and *TaCMF6* (63.27%) were significantly upregulated clusters, and they remained upregulated after vernalization; at the same time, *TaCO1* (85.71%) and *TaCO15* (66.67%) were significantly downregulated clusters remained downregulated even after vernalization. Among these clusters, *TaCMF11* and *TaCO18* may be the most relevant upregulated clusters to vernalization, and *TaCO1* may be the corresponding downregulated cluster (Fig 6, S3 Table). In general, the differentially expressed CCT genes obtained by the comparison of their expression during/after and pre-vernalization using upset plot are highly consistent with those obtained via the direct visualization of transcriptome analysis produced gene expression data using the heat map.

### Quantitative real-time PCR validates transcriptome analysis

**Fig 7:**
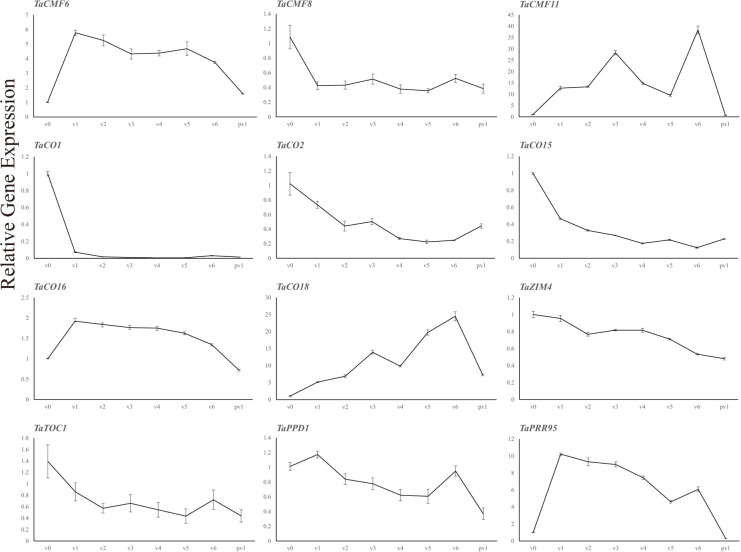
Expression profiling of CCT clusters using quantitative real-time PCR in wheat before, during, and after vernalization. The leaf samples were harvested the same as those in Fig 5. The expression level of each cluster before vernalization was used as the standard, and the relative gene expression levels during and after vernalization were calculated using the 2^-ΔΔCT^ method. The expression of CCT clusters was normalized to the housekeeping gene *TaActin*. Three biological replicates were maintained for each sample, and the deviations in gene expression level were indicated by standard error (SE).

To further validate the expression profile of vernalization-related CCT genes obtained via transcriptome analysis, quantitative real-time PCR analysis of some DEG clusters (*TaCMF6*, *TaCMF11*, *TaCO16*, *TaCO18*, *TaPRR95*, *TaCO1*, and *TaCO15*) and the most popularly studied CCT clusters (*TaZIM4*, *TaPPD1*, *TaCO2*, *TaTOC1*, and *TaCMF8*; also revealed in transcriptome analysis, but not significant differentially expressed) was conducted. As a result, *TaCMF11* (the maximum expression was 38.33 times higher than that before vernalization), *TaCO18* (24.50 times higher), *TaPRR95* (10.22 times higher), and *TaCMF6* (5.78 times higher) were significantly upregulated during vernalization, and *TaCO16* (1.92 times higher) showed slight upregulation. Surprisingly, the quantitative real-time PCR revealed that their expression levels were rapidly decreased to pre-vernalization levels after vernalization. The remaining CCT clusters analyzed by quantitative real-time PCR were found to be downregulated during vernalization (the lowest expression of *TaCO1*, *TaCO15*, *TaCO2*, *TaCMF8*, *TaPPD1*, *TaTOC1* and *TaZIM4* compared to pre-vernalization was only 0.52%, 12.44%, 22.45%, 35.56%, 36.93%, 43.49% and 48.16%, respectively) and maintained low expression levels even after vernalization. Interestingly, unlike other clusters whose expression was gradually decreased, *TaCMF8* and *TaCO1* were rapidly downregulated to low levels during vernalization (Fig 7). These results indicated that the expression patterns of these clusters were almost consistent with the results of transcriptome analysis.

## Discussion

### Genome-wide screening of CCT genes in common wheat

Plants are exposed to various external stimuli, which influence multiple processes, such as the transition from the vegetative phase to the reproductive phase and flowering. This study screened 127 distinct genes belonging to 40 clusters in common wheat against the HMM for the CCT domain obtained from Pfam v34.0 using hmmsearch command embedded in HMMER v3.0, and these genes were confirmed by the Batch CD-Search tool in NCBI [35,44]. Studies have reported 40, 41, 32, and 36 CCT genes in *Arabidopsis*, rice [8], *Brachypodium* [4], and *Medicago* [1], respectively. More CCT genes were discovered in wheat, probably because it is a hexaploidy. The majority of the CCT genes have orthologs in various species, and therefore, the wheat CCT genes were subsequently designated based on their orthologs in rice and *Brachypodium* [4,6]. Multiple sequence alignment and phylogenetic analysis of the CCT proteins revealed noticeable differences among species. For example, CMF2 was present only in rice but not in *Brachypodium*, wheat, *Setaria italica* and *Hordeum vulgare* [4]. No duplicated copies were found for *CMF13* in these four species either, but almost identical sequences of *CMF12* and *CMF13* (99.86% DNA identity and 100% cDNA identity) were found in rice. *TaCMF11* was the closest paralog of *TaCMF8*. In some works, the orthologs of *CMF11* were also named CO9 [45], but these genes should be re-designated as *CMF11* for the loss of the B-box zinc finger motif [4]. Furthermore, no ortholog of *TaCMF15* was detected in rice and *Brachypodium* [4]. *BdCMF15* is highly homologous to *OsCMF8* and *TaCMF8*, and it should be re-designated as *BdCMF8*. In addition, no rice *OsQ* homologs were found in wheat.

Furthermore, the phylogenetic analysis of CCT genes in wheat revealed eight discrete groups, consistent with the grouping based on different domains and specific CCT domain splitting (Figs 2 and 3). This study designated Groups I to III as CO/COL genes, Groups IV to VI as CMF genes, Group VII as PRR genes, and Group VIII as ZIM genes.

### Characteristics of CO/COL and CMF genes

The present study showed that many CCT proteins (100 out of 127) had only the CCT domain (CMF proteins), or harbored one or two extra B-box domains (CO/COL proteins) in wheat. Similar to the previous studies, these B-box domains were divided into two groups, B-box1 and B-box2, based on the conserved sequences and zinc-binding residue spacing. Interestingly, B-box1 was present in all of the CO/COL proteins, whereas B-box2 was present only in CO/COL proteins with two B-box domains [4,46,47]. The previous studies showed that the B-box domains generally interacted with the coiled coil domain to form a functional complex of RING, B-box, coiled-coil/Tripartite motif (RBCC/TRIM), and via this complex, CO/COL proteins functioned in various physiological processes such as photoperiod flowering, circadian rhythm, seedling photomorphogenesis, light signaling, cold/drought response, and hormone signaling [46]. Therefore, the functions of these B-box domain containing CCT genes should be further analyzed.

Phylogenetic analysis of CO/COL proteins and CMF proteins in wheat was consistent with those in *Arabidopsis*, rice, and *Brachypodium* (Fig 1) [4,33]. This classification was also consistent with that based on CCT splitting and the number of B-box domains. For example, CCT domains of Group I, II, and IV proteins were predicted to be encoded by single exons; Group III proteins split their CCT domains at the 16^th^ residue; while CCT domains of Group V and VI proteins were split after 22^th^ and 37^th^ residues, respectively. A previous study has suggested that CO/COL genes gradually lost their B-box domains, from two to one and then to none [4]. The present phylogenetic analysis of CCT proteins provided some new insight to this hypothesis: many single and double B-box-containing proteins were clustered in Groups I and III, and B-box-free Group IV proteins were found closely related to these two groups (Fig 1) [4,33].

Gene duplication is another critical event that occurs during evolution and leads to the divergence of gene families. Some recent duplications involved with CMF genes were detected in this study via multiple alignment and phylogenetic analysis. For example, *CMF6* and *CMF8* had two or three gene copies tandemly arranged in each subgenome in wheat and barley, whereas only one copy occurred in *Brachypodium* and rice [4]. These tandemly arranged genes were located near telomeres, where recombination rates are relatively high (Fig 4).

### Characteristics of PRR genes

Similar to the previous studies in *Arabidopsis*, rice, and *Brachypodium*, five PRR genes were also identified in each wheat subgenome [4,48]. This group of CCT genes harbored a Response_reg domain at the N-terminal of the proteins. The subsequent phylogenetic analysis grouped them into three clades, representing TOC1, PRR3/7, and PRR5/9 (Fig 1), which is consistent with the previous findings [49]. The expression of *Arabidopsis* PRR genes peaks by turns during photoperiod in the order of *APRR9*, *APRR7*, *APRR5*, *APRR3*, and *TOC1* [48], which form highly complex regulatory loops with CCA1/LHY and other factors [49]. The central oscillator component TOC1 represses the expression of *CCA1* and *LHY* directly [10], while promotes *CCA1* expression indirectly via CCA1 HIKING EXPEDITION (CHE) [50]. Conversely, CCA1 and LHY directly reduce *TOC1* expression, forming the feedback loop [9]. The complex feedback loops formed by PRRs and CCA1/LHY (the feedback loops formed by other PRRs are similar to those of *TOC1* and CCA1/LHY) regulate the circadian rhythm of clock genes, the expression of clock output genes, and flowering. A previous study demonstrated these via the overexpression of *APRR3* that led to longer rhythm of circadian-controlled genes under continuous white light and late flowering under LD conditions [51]. Mutation of *Ppd-H1* and *Ppd-D1* also led to the reduced photoperiod response and late flowering in barley and wheat, respectively, and this gene became the key regulator of photoperiod flowering in cereals [52,53]. Moreover, previous studies found that the *prr9prr5prr3* mutant developed shortened petioles and lengthened hypocotyls [54], and the *prr9prr7prr5* mutant was more tolerant to cold, high salinity and drought stresses than the wild type [55], indicating distinct functions of PRR genes other than flowering, such as seedling photomorphogenesis and cold/drought response.

### Characteristics of ZIM genes

The ZIM proteins were the only proteins predicted to harbor the CCT domain in the middle of the sequence, with an extra tify domain at the N-terminal and a ZnF_GATA domain at the C-terminal. The ZnF_GATA domain, with a conserved CX_2_-CX_17-20_-CX_2_C motif followed by a highly basic region, is widely distributed in fungal, animal, and plant species. Several studies have reported the critical roles of proteins harboring this domain in cell proliferation, development, and differentiation [56,57]. The tify domain, named after its most conserved TIF[F/Y]XG motif, was first discovered in the *Arabidopsis* ZIM protein. This domain has been found in proteins grouped into plant-specific families [58]. In some studies, ZIM genes were excluded from CCT genes [4], while in other studies, they were considered as CMF genes [7,8], probably due to the differences in queries used in BLAST analysis: the BLAST search against the coding sequences of all known CCT genes led to the exclusion of ZIM genes from the CCT family [4], but that against the CCT domain of Ghd7 led to the inclusion of ZIM genes [8]. The presence of the prominent CCT domains again rationalized the classification of ZIM proteins into the CCT family, and indicated their distinct role in morphogenesis and flowering. *AtZIM* was explicitly found expressed in flowers and flower buds, and its overexpression led to a hypocotyl and petiole cell elongation and leaf upward positioning [5]. The overexpression of *TaZIM-A1* also delayed flowering, because this protein could downregulate the expression of *TaCO1* and *TaFT-1* by directly binding to their promoters [6]. Similar to the previous studies, variable numbers of ZIM genes were found in different subgenomes, that is, four, three, four, and six in wheat, *Arabidopsis*, rice, and *Brachypodium* subgenome, respectively (Fig 1, S1 and S2 Tables) [56]; the divergence of ZIM1/ZIM3 and ZIM2/ZIM4 clade was also indicated in the phylogenetic analysis, in which the *Arabidopsis* ZIM proteins appeared closely related to ZIM1/ZIM3 clade (Fig 1).

### Expression profile of CCT genes during vernalization in wheat

Typically, plants need to receive and respond to various internal and external signals via highly complex pathways, such as photoperiod and vernalization, to flower at the most appropriate time. By integrating the light signals with the precise circadian clock system, CO responds to the different photoperiod, and regulates flowering [13,15]. However, the vernalization pathway is somewhat different between *Arabidopsis* and cereals. In *Arabidopsis*, *FLOWERING LOCUS C* (*FLC*) is the core flowering suppressor, which is inhibited during vernalization [59]. However, *VRN2* is the most important flowering suppressor associated with vernalization in wheat [22]. In order to clarity whether other CCT genes are related to wheat vernalization, a transcriptome analysis and a quantitative real-time PCR analysis were performed using leaf tissues before, during, and after vernalization in wheat.

The expression analysis performed via transcriptome analysis and quantitative real-time PCR revealed many low temperature-responsible CCT clusters, including upregulated and downregulated ones. *TaCMF11*, *TaCO18*, *TaPRR95*, *TaCMF6*, and *TaCO16* were significantly upregulated clusters during vernalization, but quantitative real-time PCR revealed an immediate decrease in their expression after vernalization. Many vernalization related genes have been reported to be upregulated during vernalization in wheat and *Arabidopsis*, and these genes demonstrated different expression patterns after vernalization. For example, the wheat *VRN1* showed a transient downregulation after vernalization [60], however, the wheat *VIN3-LIKE* (*VIL*) genes, together with their *Arabidopsis* ortholog *VERNALIZATION-INSENSITIVE 3* (*VIN3*), exhibited a significant and continuous downregulation after vernalization [61,62], probably depending on their functions after vernalization. These previous observations demonstrated the possible correlation between the low temperature induced CCT clusters and vernalization. However, some vernalization related genes, such as *VRN2* in wheat and *FLC* in *Arabidopsis*, were downregulated during vernalization and maintained their low expression after vernalization [22,63]. An upregulation of *VRN2* was observed after vernalization in the *vrn1*-null mutant, which flowered later than the double *vrn1*-*vrn2*-null mutant in wheat [26], indicated the importance of *VRN2* inhibition after vernalization in flowering. In this study, *TaCO1* and *TaCO15* were the significantly and continuously downregulated clusters, maintaining their low expression after vernalization. Furthermore, the quantitative real-time PCR and RNA-sequencing also revealed the downregulation of *TaCO2*, *TaCMF8*, and *TaPPD1* during and after vernalization. Interestingly, *TaCO1* and *TaCMF8* were found to be rapidly downregulated during vernalization in quantitative real-time PCR, but the same expression pattern was found only for *TaCO1* in transcriptome analysis. This is probably due to the deficiency of *TaCMF8-B2* expression data in transcriptome analysis originated from the lack of this gene in high confidence annotations of CS assembly. In summary, some low temperature induced clusters such as *TaCMF11*, *TaCO18*, *TaPRR95*, *TaCMF6*, and *TaCO16*, and vernalization downregulated clusters such as *TaCO1*, *TaCO15*, *TaCO2*, *TaCMF8*, and *TaPPD1* were obtained through transcriptome analysis and quantitative real-time PCR, and these clusters might highly correlate with flowering in wheat.

## Conclusions

The CCT family is a large family of genes closely related to circadian rhythm regulation and photoperiod flowering. The present study based on whole-genome analysis identified 127 CCT genes belonging to 40 clusters. Further analysis classified the CCT proteins into eight groups, including the CO/COL subfamily (Groups I to III), CMF subfamily (Groups IV to VI), PRR subfamily (Group VII), and ZIM subfamily (Group VIII). The transcriptome analysis revealed that several CCT gene clusters such as *TaCMF11*, *TaCO18*, *TaPRR95*, *TaCMF6*, and *TaCO16* were upregulated during vernalization, while *TaCO1*, *TaCO15*, *TaCO2*, *TaCMF8*, and *TaPPD1* were downregulated, probably related to vernalization. The definite functions of these gene clusters during vernalization still need to be further confirmed by transgenic technology. The present study provides new insights into wheat vernalization, and thus expands the possibility of increasing environmental adaptability in wheat.

## Supporting information

S1 Fig Multiple alignments of the CCT domains in wheat. (TIF)

S1 Table The detailed information of CCT genes characterized in this study. ^a^The sequences were FGENESH+ reanalyzed. ^b^The sequences were analyzed manually according to their homologous sequences.
(XLSX)

S2 Table The CCT proteins and their associated accessions in *Arabidopsis*, rice, and *Brachypodium*. (XLSX)

S3 Table The DEG list at different time points performed by transcriptome analysis. The total number of DEGs in different clusters was listed on the right.
(XLSX)

S4 Table Primers used in quantitative real-time PCR in this study. (XLSX)

S5 Table Expression data for CCT genes obtained from transcriptome analysis. The expression level was expressed as FPKM.
(XLSX)
