# Supplementary figures and images for: Genome-wide identification of CCT genes in wheat (Triticum aestivum L.) and their expression analysis during vernalization
Source: PLoS One. 2022 Jan 5;17(1):e0262147. doi: 10.1371/journal.pone.0262147 (PMC8730456; doi:10.1371/journal.pone.0262147)

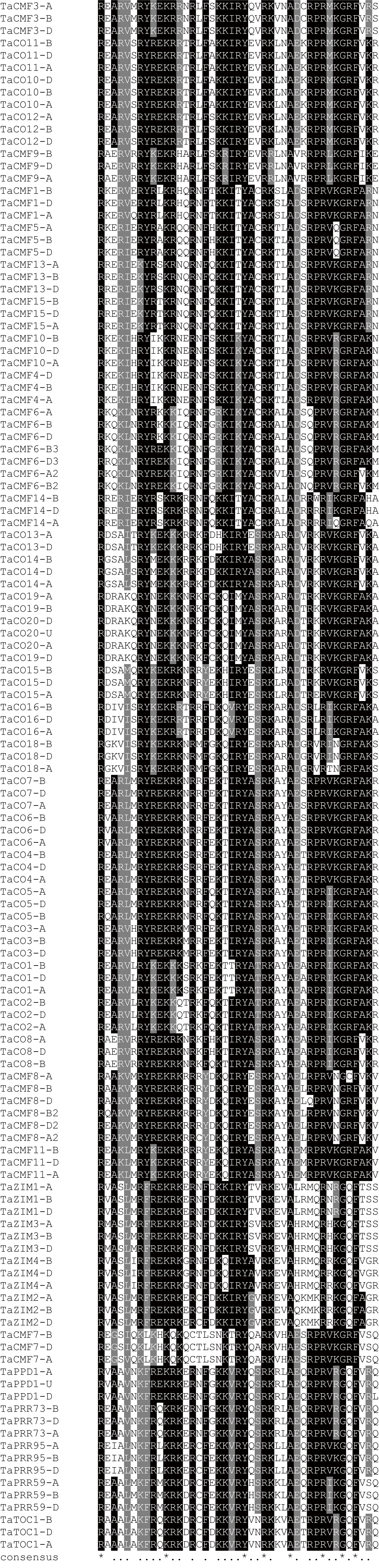

Supplement: S1 Fig — (TIF) [file pone.0262147.s001.tif]
